# Relationships between the lithology of purple rocks and the pedogenesis of purple soils in the Sichuan Basin, China

**DOI:** 10.1038/s41598-019-49687-9

**Published:** 2019-09-13

**Authors:** Shouqin Zhong, Zhen Han, Jing Du, En Ci, Jiupai Ni, Deti Xie, Chaofu Wei

**Affiliations:** 1 College of Resources and Environment, Southwest University, Chongqing, 400715 China; 2 State Cultivation Base of Eco-agriculture for Southwest Mountainous Land, Chongqing, 400715 China; 3 Key Laboratory of Arable Land Conservation (Southwest China), Ministry of Agriculture, Chongqing, 400715 China; 4 Rural Energy and Environment Agency of Chongqing, Chongqing, 401121 China

**Keywords:** Geomorphology, Sedimentology, Geophysics

## Abstract

Classified as Regosols in the Food and Agriculture Organization (FAO) Taxonomy, purple soils formed from purple rocks and are mainly distributed in the Sichuan Basin of southwestern China. A number of studies have focused on the soil water, nutrients, texture and erosion of purple soils. This study was conducted to understand the lithological features of the related purple rocks and their effects on the pedogenesis of purple soils in the Sichuan Basin. The results showed the following: due to variability in the paleoenvironment, purple rocks mainly consist of sandstone and mudstone with various stratal thicknesses and various particle sizes. The lithology of the purple rocks leads the purple soils have an obvious inheritance from their parent rocks. An apparent purple color and numerous rock fragments derived from the purple parent rock are observed throughout the profile, with no clear soil stratification. The particle size contents of the purple soils are closely related to those of their parent rocks. The clay-sized fractions in the purple soils are generally dominated by illite, vermiculite, chlorite, and montmorillonite with little quartz and with or without kaolinite, which is generally the same as that in the parent purple rocks. In addition, the purple soils are characterized by obvious inherited mineralogy, chemical composition, pH value, OM content and nutrient content. Therefore, the diagenetic environment determined the lithology of the purple rock, and the lithology of the purple rock determined the pedogenic characteristics of the purple soil to some extent. Purple soils are characterized by rapid physical weathering and pedogenetic processes and slow chemical pedogenetic processes.

## Introduction

Geological processes create conditions for soil development^1–3^. The concept of the soil-forming factor is one of the earliest and most important issues in soil science. This concept defines soil as a component of ecosystems that must be characterized in terms of both geological substrate and biological input^4^. Soil formation is driven by gradients in chemical and physical potential as the earth’s atmosphere and biosphere interact with rocks and minerals^2,5^. The lithology of the parent materials determines a soil’s physical and mineralogical properties^1,2,6–8^. As a synergetic process, pedogenesis essentially consists of the generation, selection, accumulation and differentiation of solids produced in the course of biotic and abiotic processes occurring within a soil body^9^. Lin (2010) found that the dissipating and organizing processes of pedogenesis were consistent with the theory of dissipative structure, which well explained the genesis and evolution of various soils^10^. Soil formation is usually envisaged as acting from the surface downwards^11^, but the accumulation of sediment and soil formation is simultaneous in many environments^12^. Geogenic vs. pedogenetic is an indistinct and partly artificial distinction, as some processes such as weathering could be considered under both headings^13^. Mirabella *et al*. (1996) performed petrographic and mineralogical analyses on parent rock materials and secondary clay minerals, respectively, and the results indicated that there was a close connection between weathering and pedogenetic processes^14^. In some special environments, e.g., mountainous areas in the humid tropics and subtropics, Gracheva (2011) investigated the role of time and erosion in soil diversity with different properties of parent rocks and weathered mantle^15^.

Purple soil is mainly distributed in the Sichuan Basin of southwestern China and has developed from purple rocks or their rapid weathering products, thus inheriting many of the characteristics of the parent materials or rocks^16–20^. This type of soil is classified as a Regosol in the Food and Agriculture Organization (FAO) Taxonomy and as an Entisol in United States Department of Agriculture (USDA) Taxonomy^3,17^. Purple rocks are broken up into rock fragments by fast physical weathering and are generally considered a nutrient reservoir because of their high concentrations of mineral nutrients, especially phosphorus and potassium^17^. Nutrients released from purple rock fragments in the weathering process is a major source of nutrients in purple soils. Due to their high mineral nutrient contents, purple soils are fertile, and the cooccurrence of abundant rainfall and a warm climate is favorable for crop growth, which makes the Sichuan Basin one of the most densely populated agricultural regions in China. The density of the population in Sichuan Basin ranges between 100 and 800 people/km^2^, and arable land resources are very limited, with some slopes over 35° still under cultivation^18,21^. The depth of the soil layer for in upland purple soil areas is less than 50 cm, which makes it easily eroded by rainfall and runoff. The weighted mean net soil loss from upper and lower subfields has been estimated to be 48.7 t ha^−1^ year^−1^ to 16.9 t ha^−1^ year^−1^, respectively^21^. For purple soil, more than 0.3 cm of surface soil is eroded every year, and serious soil erosion has caused extensive land degradation that endangers sustainable agriculture development^22^. Therefore, the study of the mechanisms of accelerated transformation of purple rock fragments into soil materials is very important for agricultural production in the Sichuan Basin^3,23^. Furthermore, the soil water storage capacity and infiltration capacity of purple soil are low, which leads to a high runoff coefficient, representing a major cause of soil erosion in the Sichuan Basin^24^. However, desertification has not occurred in the purple soil areas in the Sichuan Basin. To understand the mechanism of soil erosion and keep the fertile soil sustainable for agriculture, it is necessary to understand the soil genesis from purple rock and the related characteristics.

Therefore, a better understanding of the lithological features of purple rock and its effect on the pedogenesis of purple soil is necessary in order to develop sustainable agriculture in the Sichuan Basin of southwestern China. This paper takes the Sichuan Basin as the study area, explores the variations in paleoenvironments during the Mesozoic Era, analyzes the lithology of 6 typical purple rocks and the properties of 6 purple rocks-derived soils, and determines the characteristics of the pedogenetic process and the relationships between lithology and pedogenesis.

## Materials and Methods

### Study area

**Figure 1:**
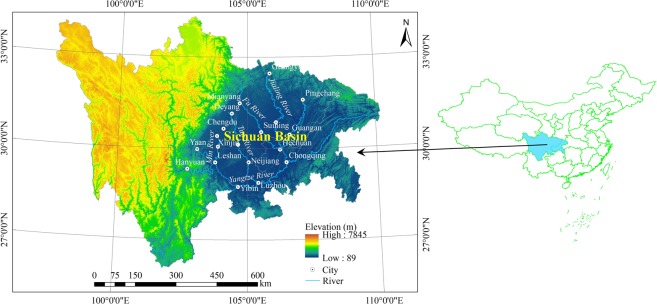
Location of the study area.

The Sichuan Basin (30°30′N, 105°30′E) is a lowland region in southwestern China, and it is surrounded by upland regions and mountains (Fig. 1). The Tibetan Plateau lies to the west and northwest, the Yungui Plateau extends to the south and southeast and to the east lie several hundred miles of mountains cut by the eastward-flowing Yangtze River. The climate is classified as subtropical humid monsoon with an annual average temperature of 14–19 °C and an evaporation of 920–1,570 mm. The annual average rainfall is high (approximately 1,000–1,400 mm yr^−1^), but the precipitation is unevenly distributed throughout the year, with the majority occurring in the period from April and September. The fertile purple soils are largely exposed in extensive areas of reddish sandstone and purple mudstone, leading to the “Red Basin” nickname for the region.

### Soil sampling

According to the geology of Sichuan Province^25^, the purple rock strata of the Triassic to Cretaceous system mainly consist of the Cretaceous Jiaguan Formation (K_2_*j*) and Chengqiangyan Group (K_1_*c*) and the Jurassic Penglaizhen Formation (J_3_*p*), Suining Formation (J_3_*s*), Shaximiao Formation (J_2_*s*), and Ziliujing Formation (J_1__-__2_*z*). Samples from the above formations have been selected to represent the wide range of principal purple rocks and principal purple soils in the Sichuan Basin^17^.

In total, 227 soil samples from a depth of 0–20 cm were randomly collected, including 32 samples of the red purple soil developed from the purple rocks of the Jiaguan Formation (K_2_*j*), 27 samples of the yellowish red purple soil developed from the Chengqiangyan Group (K_1_*c*), 43 samples of the brown purple soil developed from the Penglaizhen Formation (J_3_*p*), 70 samples of the reddish brown purple soil developed from the Suining Formation (J_3_*s*), 35 samples of the grayish brown purple soil developed from the Shaximiao Formation (J_2_*s*), and 20 samples of the dark purple soil developed from the Ziliujing Formation (J_1__-__2_*z*). Each soil sample is a composite consisting of four to six subsamples. All soil samples were first air-dried and then sieved through a 2 mm screen after removal of visible pieces of plant debris and stones. Finally, the soil samples were mixed and stored in a 4 °C environment prior to laboratory analysis. To determine the geochemical inheritance of the purple soils, five representative soil profiles were selected from each soil formation and sampled for laboratory analysis. Each purple rock formation has 5 samples, and the data of rocks were obtained on parent rocks or partly weathered parent rocks from the C horizon or BC horizon of soil profiles. The uniformity of the rock profiles was observed from the C horizon or BC horizon of soil profiles and the exposed rock strata around the sampling sites.

### Laboratory methods

The particle size distribution of parent rock samples was measured according to the analysis method for particle size of clastic rocks^26^. According to the methods of soil and plant analysis^27^, the particle size distribution of soil samples was tested by the pipette method with pretreatment for the removal of organic matter (OM) using H_2_O_2_ with Na hexametaphosphate as the dispersant agent. The CaCO_3_ content in the soil and rock was determined with a Dietrich-Fruehling calcimeter. Cation exchangeable capacity (CEC) of the soil and rock was determined by leaching air-dried soil samples with 1.0 mol L^−1^ NH_4_OAc solution. The pH was measured using a pH meter.

The OM content of the soil and rock was tested by oxidation with potassium dichromate (K_2_Cr_2_O_7_) and titration of excess dichromate with ammonium ferrosulfate [(NH_4_)_2_FeSO_4_]. Total nitrogen (TN) of the soil and rock was determined by the Kjeldahl method using concentrated H_2_SO_4_, K_2_SO_4_ and HgO to digest the sample, and the available nitrogen (AN) of the soil and rock was determined via 1.0 mol L^*−*1^ NaOH extraction and distilling. Total phosphorus (TP) of the soil and rock was measured by colorimetry after digestion with HClO_4_ + H_2_SO_4_, and the available phosphorus (AP) was measured by colorimetry after extraction with 0.5 mol L^*−*1^ NaHCO_3_ (pH = 8.5). The total potassium (TK) of the soil and rock and available potassium (AK) of the soil and rock was measured by a flame photometer using the NaOH fusion method and extraction with NH_4_OAc, respectively.

The total Si, Al, and Fe in the clay fraction of soil and rock were tested after digestion with HF and H_2_SO_4_. The Al and Fe were measured by atomic absorption/emission spectrometry, whereas total Si was measured by atomic absorption spectrometry (AAS) after fusion with sodium carbonate^28^. The minerals in the clay fraction of soil and rock was tested for all horizons by X-ray diffraction (XRD) using monochromated CuKα radiation on oriented samples after being Mg-saturated, treated with ethylene glycol and heated to 350 °C. The diffractograms were interpreted according Brindley and Brown (1980)^29^.

## Results

### Variations in paleoenvironments during the mesozoic era in the sichuan basin

The parent material of purple soil is purple sedimentary rock of the Mesozoic Erathem. Due to variations in the paleoenvironment during the Mesozoic Era, many purple sedimentary rocks with various lithological features formed in the Sichuan Basin, most of which were lacustrine facies sedimentary rocks deposited in the Jurassic Period and the Cretaceous Period. Only the dark purple shale of the Feixiangguan Formation (T_1_*f*) of the Triassic System is the sedimentary rock of marine facies.

**Figure 2:**
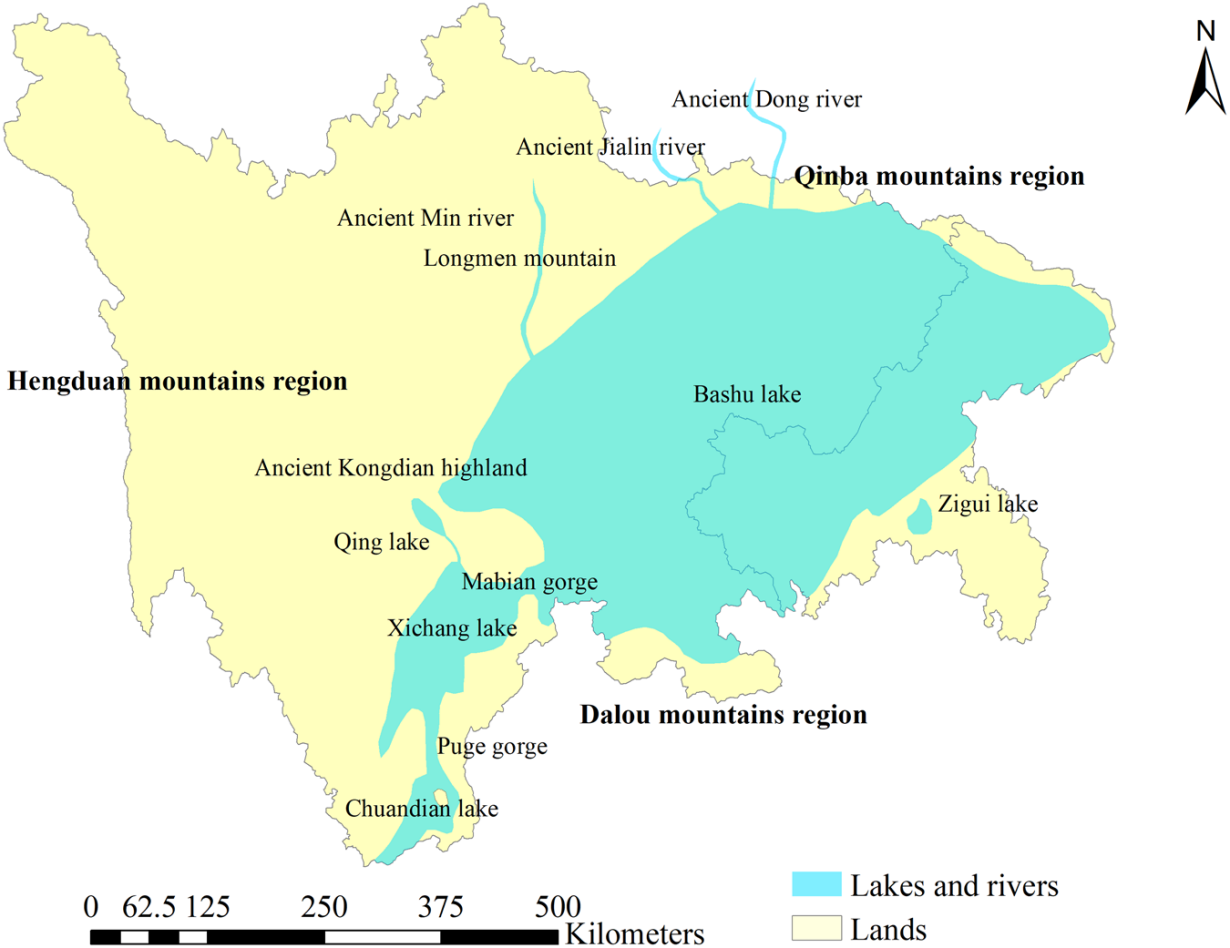
Schematic diagram of the Jurassic palaeoenvironment of the Sichuan Basin.

Starting in the early Jurassic period, hot and arid conditions prevailed in the Sichuan Basin. The mountainous region to the west of the basin and its marginal hills were eroded by water^30^. Purple-red sediments with a thickness of more than 4,000 m were deposited in Bashu Lake and Xichang Lake (Fig. 2), and forming the purple sedimentary rocks in the Sichuan Basin. Because of the effect of the Yanshan Movement in the Late Jurassic period, parallel mountain range stretching from the north to the east appeared in the eastern Sichuan, and a mountainous region to the southeast of Sichuan Basin was uplifted. As a result, Bashu Lake retreated to the northwest of Sichuan Basin.

**Figure 3:**
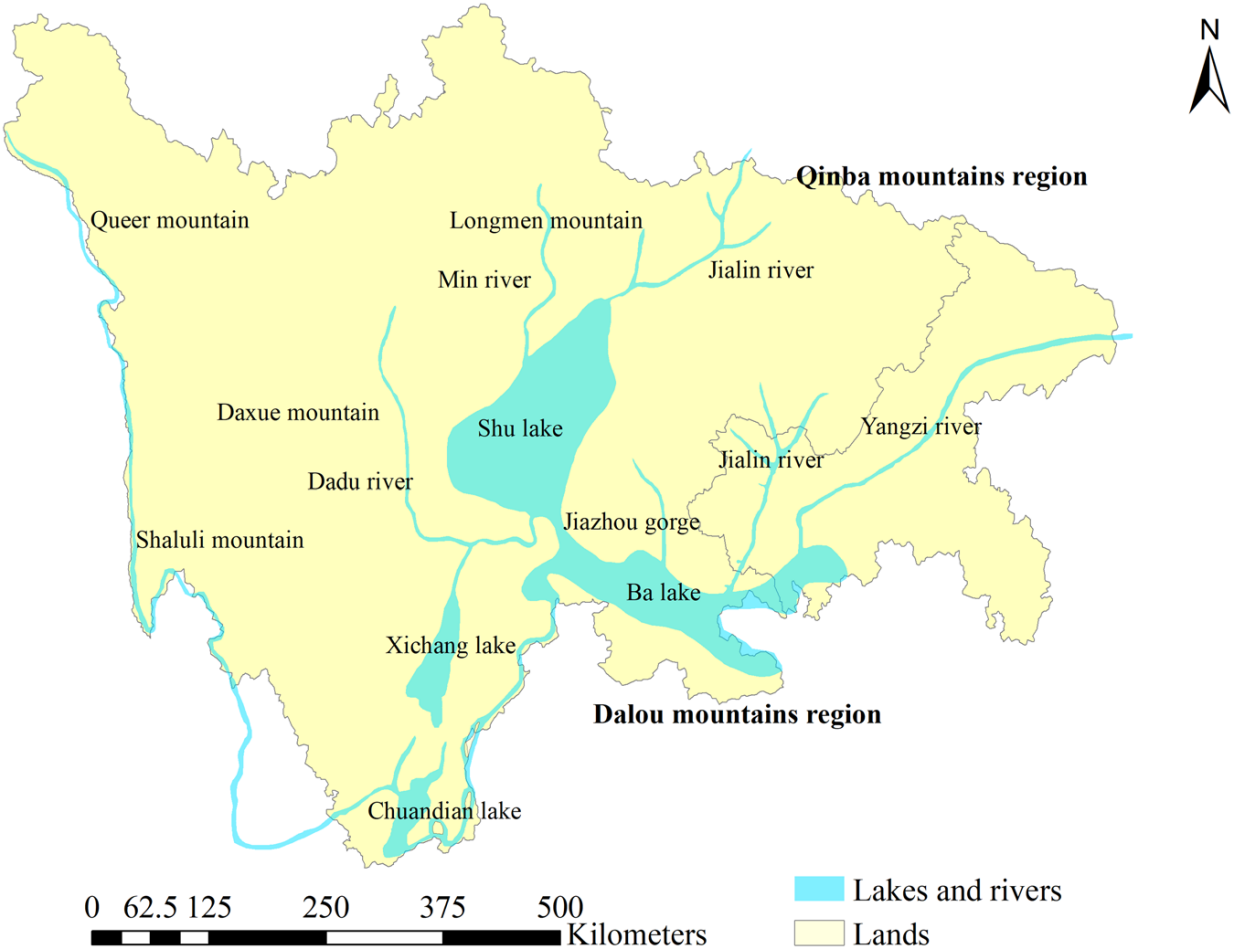
Schematic diagram of the Cretaceous palaeoenvironment of the Sichuan Basin.

In the Cretaceous period, areas in the southeastern and the central Sichuan Basin were lifted, while the depression in the northern and northwestern basin became increasingly deep^30^. Bashu Lake further shrank to only 20,000 km^2^ and is referred to at this time as Shu lake (Fig. 3). The sediment eroded from the mountainous regions of western Sichuan was transported to Bashu Lake via Guangyuan, Yanting, and Guanxian. This material was source material for the purple sedimentary rocks in the Chengqiangyan Group of the Cretaceous system in the Sichuan Basin. During the late Cretaceous period, the areas of the northern basin and Yunnan-Guizhou plateau rose progressively, and Shu Lake retreated southward to Anxian. The depocenter of this lake was situated between Yaan and Leshan, and the deposited material was transformed into the purple sedimentary rocks of the Cretaceous Guankou Formation and Jiaguan Formation in the Sichuan Basin. The approximately 20,000 km^2^ Ba Lake formed in front of Dalou Mountain and west to Qijiang and north to Gejiang, Luzhou, and Zigong, and material eroded from Dailou Mountain was deposited in this lake, forming deposits up to 2,000 m thick, which became the purple sedimentary rocks of the Late Cretaceous Jiaguan Formation in the Sichuan Basin.

Due to the variations in the paleoenvironment, the purple rocks mainly consist of sandstone and mudstone, and the units vary inthickness and in particle sizes. The basic colors of these rocks are brick red, brownish red, reddish brown, and grayish purple. The materials forming these purple sedimentary rocks originated from the mountainous region around Sichuan Basin. The different types of rocks (and their weathering fragments) are interbedded and were alternately deposited and diagenetically altered in paleolakes in the Sichuan Basin.

### Lithology of purple rocks

#### Uniformity of the rock profiles

Different purple rocks have different profile features. (1) The rocks of the Cretaceous Jiaguan Formation (K_2_*j*), deposited and diagenetically altered in rivers and lakes under increasingly arid weather, are relatively uniform. These rocks consist of feldspathic sandstone with fine- to medium-grained particles, a color ranging from purplish red to light yellow. The rocks are characterized by a high hygroscopicity and water permeability, easy eluviation and fast acidification. (2) The rocks of the Cretaceous Chengqiangyan Group (K_1_*c*), deposited and diagenetically altered in the rivers and alluval plains under an arid and hot climate, consist of thick feldspathic sandstone strata with thin-bedded brownish red, purplish red, or dark purple silty sandstone and mudstone strata. These rocks are characterized by a high hygroscopicity and thermal diffusion, with moderate erosion resistance. (3) The rocks of the Jurassic Penglaizhen Formation (J_3_*p*), deposited and diagenetically altered in a shallow lake or near the lake shore under hithly arid conditions, consist of a thick layer of purplish red mudstone and purplish red sandstone. The rocks are characterized by a high porosity with many wide crevices. (4) The rocks of the Jurassic Suining Formation (J_3_*s*), deposited and diagenetically altered in large shallow lakes under arid and hot conditions, are thick-bedded mudstones with thin- to medium-bedded silty sandstone and, are bright red or brownish red. The rocks are characterized by a low porosity and much narrow crevices. (5) The rocks of the Jurassic Shaximiao Formation (J_2_*s*), deposited and diagenetically altered on a river floodplain or in a near-shore environment in a shallow lake under relatively humid conditions, are composed of grayish yellow to grayish white mudstone interbedded with thick-bedded sandstone that is grayish purple, gray, purplish gray, grayish purple or yellow in color. These rocks are characterized by a high porosity. (6) The rocks of the Jurassic Ziliujing Formation (J_1__-__2_*z*) were deposited and diagenetically altered in lakes or rivers under mild humid conditions. The lithofacies of these rocks are somewhat complex: the upper portion is composed of grayish green or dark purplish red mudstone; the middle is composed of thin- to medium-bedded gray carbonate rock, muddy carbonate rock mingled with purplish red or grayish purple mudstones; and the lower portion is composed of purplish red mudstones mixed with thin- to medium-bedded yellowish green silty sandstone or fine-grained quartz sandstones. These rocks are characterized by a low porosity and very fine crevices. Therefore, to the variability of paleoenvironment, the diagenetic environment determined the lithology of the purple rocks.

#### Particle size distribution of the rocks

Purple rock particle size distribution depends greatly on the rocks’ diagenetic environment (Table 1). The sand contents of the purple rocks range between 35.83% and 58.69%, and tend to be higher in the red purple rocks (58.69%) of the Cretaceous Jiaguan Formation (K_2_*j*), grayish brown purple rocks (48.11%) of the Jurassic Shaximiao Formation (J_2_*s*) and dark purple soils (47.54%) of the Jurassic Ziliujing Formation (J_1__-__2_*z*) and lower in other purple rocks (35.83–39.38%). The coarse sand contents in the purple soils are less than 5.00% except in the dark purple rocks (10.81%) and grayish brown purple rocks (7.43%). Compared to the sand contents, the clay contents of the purple rocks exhibit the opposite pattern: the clay contents of the red purple rocks (14.56%) and grayish brown purple rocks (15.33%) are much lower than those of the other rocks (17.38–18.79%).

**Table 1:** The particle size distribution of the purple rocks.

Rock		*n*	Particle size (Mean ± SD, %)			
			Coarse sand (2–0.2 mm)	Fine sand(0.2–0.02 mm)	Silt(0.02–0.002 mm)	Clay (<0.002 mm)
K_2_*j*	Red purple rock	5	1.27 ± 0.34	57.42 ± 2.93	26.75 ± 1.98	14.56 ± 1.29
K_1_*c*	Yellowish red purple rock	5	0.32 ± 0.25	39.06 ± 2.35	43.24 ± 2.51	17.38 ± 2.28
J_3_*p*	Brown purple rock	5	0.58 ± 0.28	35.25 ± 4.21	45.38 ± 2.78	18.79 ± 2.35
J_3_*s*	Reddish brown purple rock	5	2.19 ± 0.54	33.83 ± 1.66	46.48 ± 3.61	17.50 ± 1.66
J_2_*s*	Grayish brown purple rock	5	7.43 ± 1.73	40.68 ± 3.34	36.56 ± 1.72	15.33 ± 1.39
J_1__-__2_*z*	Dark purple rock	5	10.81 ± 1.39	36.73 ± 1.27	34.25 ± 1.54	18.21 ± 2.32

#### Mineralogy and chemical composition of rocks’ clay fractions

**Figure 4:**
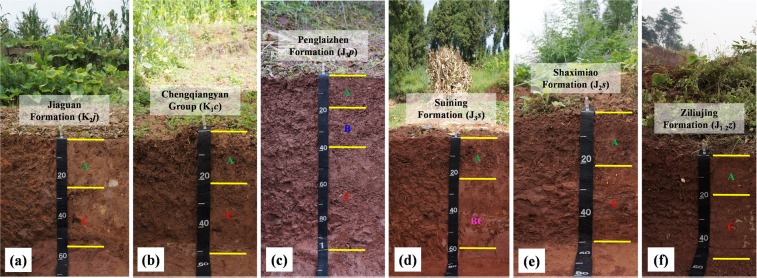
Soil profiles developed from different purple rocks. A, B, BC, and C denote the A horizon, B horizon, BC horizon, and C horizon, respectively.

**Figure 5:**
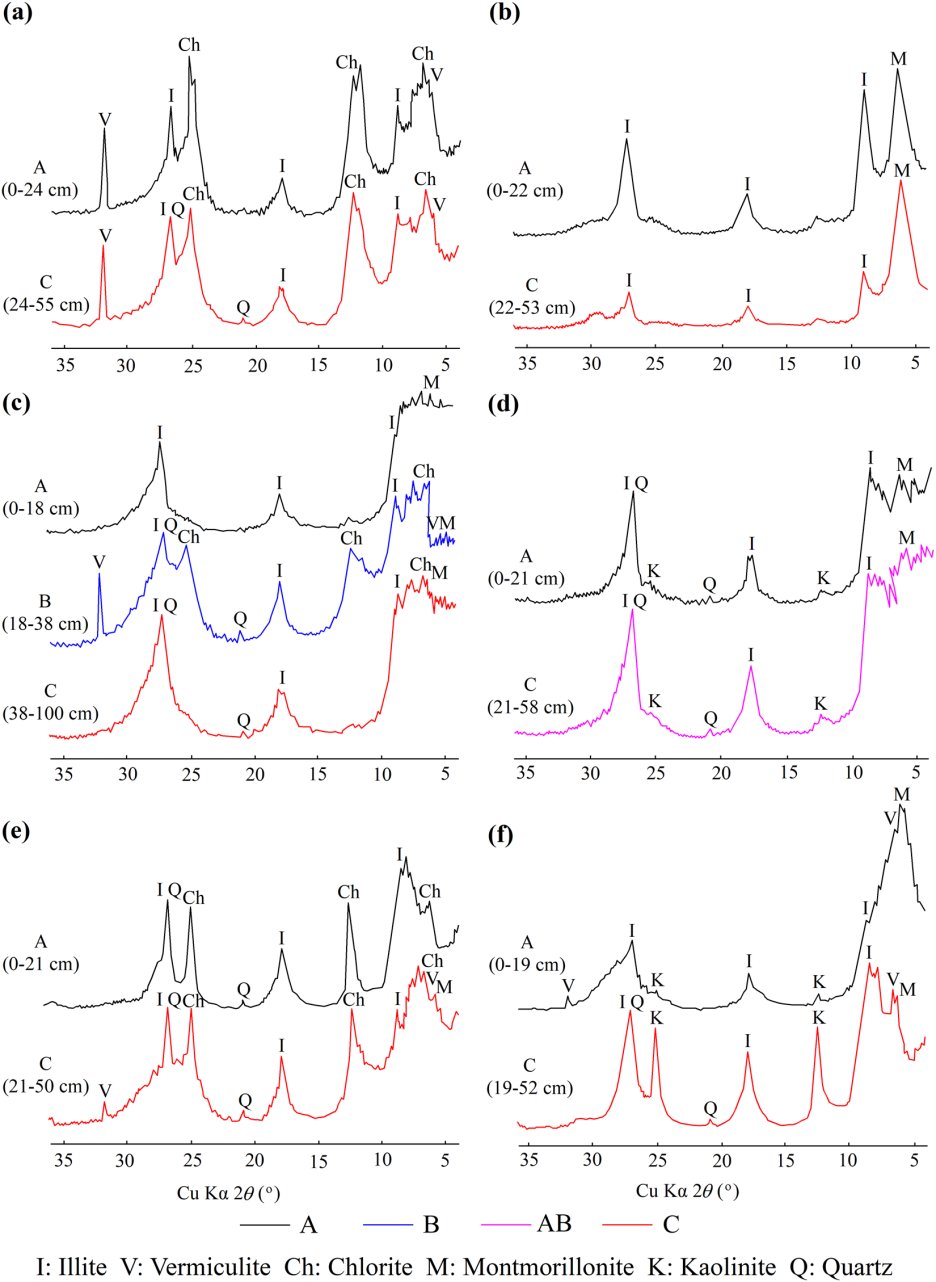
X-ray diffraction diagrams of the clay fractions in the purple soils developed from the rocks of K_2_*j* (**a**), K_1_*c* (**b**), J_3_*p* (**c**), J_3_*s* (**d**), J_2_*s* (**e**) and J_1-2_*z* (**f**).

To determine geochemical inheritance of purple soils, the mineralogy and chemical composition of the rocks’ clay fractions were obtained from the C horizon or BC horizon of representative soil profiles (Fig. 4). The clay-sized fraction in the purple rocks is generally dominated by illite (2*θ* at 8.84°, 17.73°, 26.76°), vermiculite (2*θ* at 6.14°, 31.84°), chlorite (2*θ* at 6.22°, 12.38°, 25.01°), and montmorillonite (2*θ* at 5.70°~5.85°), with little quartz (2*θ* at 20.90°, 26.65°) and with or without kaolinite (2*θ* at 12.38°, 24.93°) (Fig. 5). The SiO_2_/Al_2_O_3_, SiO_2_/Fe_2_O_3_ and SiO_2_/R_2_O_3_ ratios of the clay fraction in the purple rocks are shown in Table 2. The SiO_2_/Al_2_O_3_ ratio of the clay fraction in the purple rocks varies from 3.61 to 4.70 and is higher in the brown purple rocks (4.70) and grayish brown purple rocks (4.60), than in the red purple rocks (3.61) and dark purple rocks (4.00). The SiO_2_/Fe_2_O_3_ and SiO_2_/R_2_O_3_ ratios of the clay fraction in the purple rocks range from 12.59 to 18.20 and from 2.81 to 3.70, respectively. The CEC in the purple rocks varies from 15.25 cmol_c_ kg^−1^ to 20.39 cmol_c_ kg^−1^, and the exchangeable cations in the purple rocks are regarded as a direct source of soil nutrients.

**Table 2:** SiO_2_/Al_2_O_3_, SiO_2_/Fe_2_O_3_ and SiO_2_/R_2_O_3_ ratios of the clay fraction in the purple rocks.

Rock		*n*	SiO_2_ (%)	Al_2_O_3_ (%)	Fe_2_O_3_ (%)	SiO_2_/Al_2_O_3_	SiO_2_/Fe_2_O_3_	SiO_2_/R_2_O_3_	CEC (cmol_c_ kg^−1^)
K_2_*j*	Red purple rock	5	48.55 ± 1.17	22.84 ± 0.48	10.28 ± 0.23	3.61 ± 0.12	12.59 ± 0.36	2.81 ± 0.07	15.25 ± 2.12
K_1_*c*	Yellowish red purple rock	5	51.25 ± 1.84	19.57 ± 0.37	8.15 ± 0.35	4.45 ± 0.11	16.77 ± 0.43	3.52 ± 0.12	16.58 ± 2.35
J_3_*p*	Brown purple rock	5	58.56 ± 2.36	21.20 ± 0.44	8.93 ± 0.22	4.70 ± 0.14	17.49 ± 0.38	3.70 ± 0.13	20.39 ± 1.29
J_3_*s*	Reddish brown purple rock	5	52.88 ± 3.11	20.23 ± 0.32	7.75 ± 0.17	4.44 ± 0.15	18.20 ± 0.45	3.57 ± 0.09	17.63 ± 2.28
J_2_*s*	Grayish brown purple rock	5	55.19 ± 1.28	20.40 ± 0.70	9.48 ± 0.36	4.60 ± 0.13	15.52 ± 0.39	3.55 ± 0.10	15.82 ± 2.10
J_1__-__2_*z*	Dark purple rock	5	51.18 ± 2.33	21.74 ± 0.28	9.00 ± 0.29	4.00 ± 0.08	15.16 ± 0.24	3.17 ± 0.06	16.17 ± 1.72

#### Calcium carbonate content and pH value of the rocks

The calcium carbonates in the purple rocks in the Sichuan Basin mainly originates from the diagenetic environment (Table 3). The carbonate contents of the brown purple rock and reddish brown purple rock are high, with the values of 6.35% and 6.87%, respectively, and the associated pH values are approximately 8.1 and 8.2, respectively. The carbonate content of yellowish red purple rock is slightly lower than the above two purple rocks, with a value of 5.68%, and the associated pH value is approximately 7.9. The calcium carbonate contents of the grayish brown purple rock and dark purple rock are 1.29% and 1.03% respectively, and the associated pH values are about 7.2 and 7.3, respectively. The calcium carbonate is not present in the red purple rock, and the associated pH value is approximately 5.8. The large differences in calcium carbonate contents and pH values among the purple rocks result from the material sources and diagenetic environment of purple rocks.

**Table 3:** Calcium carbonate contents and pH values of the purple rocks.

Rock		*n*	Calcium carbonate content (%)	pH
K_2_*j*	Red purple rock	5	—	5.8 ± 0.1
K_1_*c*	Yellowish red purple rock	5	5.68 ± 1.12	7.9 ± 0.2
J_3_*p*	Brown purple rock	5	6.35 ± 1.23	8.1 ± 0.1
J_3_*s*	Reddish brown purple rock	5	6.87 ± 0.85	8.2 ± 0.1
J_2_*s*	Grayish brown purple rock	5	1.29 ± 0.67	7.2 ± 0.2
J_1__-__2_*z*	Dark purple rock	5	1.03 ± 0.28	7.3 ± 0.2

#### Organic matter and nutrient contents of the rocks

The OM and nutrient contents of the purple rocks are shown in Table 4. The OM contents of the purple rocks vary from 2.1 g kg^−1^ to 3.6 g kg^−1^. The OM contents of the yellowish red purple rock, grayish brown purple rock and dark purple rock are higher than 3.0 g kg^−1^. The OM contents of the red purple rock and brown purple rock are less than the three purple rocks above, with values of approximately 2.7 g kg^−1^ and 2.4 g kg^−1^, respectively. The OM content of the reddish brown purple rock is the lowest, with a value of approximately 2.1 g kg^−1^. The nitrogen content is generally low in the purple rocks, with the TN ranging from 0.35 to 0.54 g kg^−1^ and the AN ranging from 32 to 45 mg kg^−1^. The phosphorus and potassium contents are generally high in the purple rocks. The TP and TK range from 0.26 to 0.57 g kg^−1^ and from 7.0 to 11.8 g kg^−1^, respectively, and the AP and AK range from 2.7 to 5.3 mg kg^−1^ and from 28 to 69 g kg^−1^, respectively. Therefore, the purple rocks are characterized by insufficient nitrogen and abundant phosphorus and potassium.

**Table 4:** Organic matter and nutrient contents of the purple rocks.

Rock		*n*	OM	TN	AN	TP	AP	TK	AK
			(g kg^−1^)	(g kg^−1^)	(mg kg^−1^)	(g kg^−1^)	(mg kg^−1^)	(g kg^−1^)	(mg kg^−1^)
K_2_*j*	Red purple rock	5	2.7 ± 1.4	0.35 ± 0.22	33 ± 12	0.30 ± 0.17	3.9 ± 1.4	8.2 ± 3.3	28 ± 15
K_1_*c*	Yellowish red purple rock	5	3.6 ± 1.0	0.52 ± 0.07	42 ± 15	0.26 ± 0.11	3.5 ± 0.8	7.0 ± 2.9	36 ± 12
J_3_*p*	Brown purple rock	5	2.4 ± 1.3	0.47 ± 0.13	37 ± 16	0.57 ± 0.25	4.4 ± 1.5	11.8 ± 2.4	69 ± 25
J_3_*s*	Reddish brown purple rock	5	2.1 ± 0.6	0.41 ± 0.14	32 ± 13	0.41 ± 0.16	2.7 ± 0.7	9.5 ± 4.8	60 ± 19
J_2_*s*	Grayish brown purple rock	5	3.3 ± 1.2	0.43 ± 0.11	36 ± 10	0.38 ± 0.13	5.3 ± 1.6	8.3 ± 2.0	55 ± 21
J_1__-__2_*z*	Dark purple rock	5	3.2 ± 0.7	0.54 ± 0.15	45 ± 12	0.33 ± 0.07	4.2 ± 0.5	7.2 ± 2.5	44 ± 14

Note: OM, organic matter; TN, total nitrogen; AN, available nitrogen; TP, total phosphorus; AP, available phosphorus; TK, total potassium; AK, available potassium.

### Properties of purple soils

#### Uniformity of the soil profiles

Significant soil stratification is not observed in the soil profile, and an apparent purple color, derived from the purple parent rock, is observed throughout the profile. Except for a few colloidal particles or coatings, there are usually no new formations in the soils. The boundaries between soil layers are generally not as distinct as those in red soils^31^. The B horizon or alluvial layer, is very difficult to distinguish from the parent material layer by the naked eye, and they are commonly united as BC horizon. In the field, the soil horizons are differentiated only by their aggregates, degree of compaction, rock fragments, and root distribution.

Soil development on steep mountainous topography generally leads to thin and undifferentiated pedons. Purple soil has developed on hilly and mountainous topography, in addition to flat areas, so heavy soil erosion has resulted in thin and undifferentiated pedons (Fig. 4). The depth of a purple soil pedon is commonly less than 100 cm, most ranging from 30 cm to 50 cm. The soil profiles are generally characterized as A-C and A-CR. Sometimes A-B-C could be found in clayey purple soils.

#### Particle size distribution of the soils

Numerous rock fragments exist in the purple soils because of the fast physical weathering of the purple rocks. Highly gravelly and stony purple soils account for 20% of the total purple soils. The majority of rock fragments are unweathered to partially and originate from purple rocks, and the remaining fraction of rock fragments are calcareous concretions formed in the pedogenic process and rock weathering process. The calcareous concretions are mainly present in the soils developed from the purple rocks of the Jurassic Suining Formation (J_3_*s*) and Shaximiao Formation (J_2_*s*). The rock fragment contents vary from 5% to 50% in different purple soils, with the lowest in grayish brown purple soil and the highest in dark purple soils. For a given parent rock lithology, the rock fragment content increases with increasingly slope gradient on hillslopes. Furthermore, in a given area, the rock fragment content in the soil varies greatly, ranging from 1% to 30%, and increases with increasing soil depth in the profiles.

The soil particle size distribution refers to the proportions of coarse sand (2–0.2 mm), fine sand (0.2–0.02 mm), silt (0.02–0.002 mm), and clay (<0.002 mm) in the soil mass, as defined by the International Soil Science Society (ISSS)^32^. The purple soil particle distribution depends greatly on the soils’ parent rocks (Table 5). The sand contents range between 36.66% and 61.28%, and tend to be higher in the red purple soils (61.28%) developed from the purple rocks of the Cretaceous Jiaguan Formation (K_2_*j*), grayish brown purple soils (51.26%) developed from the purple rocks of the Jurassic Shaximiao Formation (J_2_*s*) and dark purple soils (47.60%) developed from the purple rocks of the Jurassic Ziliujing Formation system (J_1__-__2_*z*), and lower in other purple soils (36.66–38.97%). The coarse sand contents of the purple soils mostly are less than 5.00%, except in the dark purple soils (21.13%) and grayish brown purple soils (13.33%). The clay contents of the purple soils exhibit the opposite pattern than the sand contents, and are much lower in the red purple soils (16.38%) and grayish brown purple soils (18.36%) than in the other soils (21.81–23.32%).

**Table 5:** The particle size distribution of the purple soils from various purple rocks.

Rock	Soil	*n*	Particle size (Mean ± SD, %)			
			Coarse sand (2–0.2 mm)	Fine sand (0.2–0.02 mm)	Silt (0.02–0.002 mm)	Clay (<0.002 mm)
K_2_*j*	Red purple soil	32	4.28 ± 2.26	57.00 ± 21.03	22.34 ± 9.11	16.38 ± 9.24
K_1_*c*	Yellowish red purple soil	27	1.47 ± 1.21	37.50 ± 8.79	39.22 ± 9.86	21.81 ± 5.74
J_3_*p*	Brown purple soil	43	1.84 ± 1.24	34.82 ± 11.87	40.02 ± 8.16	23.32 ± 9.28
J_3_*s*	Reddish brown purple soil	70	4.95 ± 4.06	32.00 ± 15.45	40.84 ± 10.41	22.21 ± 8.86
J_2_*s*	Grayish brown purple soil	35	13.33 ± 15.48	37.93 ± 9.33	30.38 ± 6.65	18.36 ± 7.83
J_1__-__2_*z*	Dark purple soil	20	21.13 ± 14.08	26.47 ± 9.12	29.41 ± 9.94	22.99 ± 12.09

#### Mineralogy and chemical composition of soils’ clay fractions

Clay minerals strongly influence the main physical and chemical properties of soils. Therefore, questions concerning the origin, distribution and formation of these minerals have become prominent issues in soil research^33^. The clay-sized fraction in the purple soils is analyzed using XRD (Fig. 5) and one can see that they are generally dominated by illite (2*θ* at 8.84°, 17.73°, 26.76°), vermiculite (2*θ* at 6.14°, 31.84°), chlorite (2*θ* at 6.22°, 12.38°, 25.01°), and montmorillonite (2*θ* at 5.70°~5.85°), with little quartz (2*θ* at 20.90°, 26.65°) and with or without kaolinite (2*θ* at 12.38°, 24.93°) because of their low degree of weathering. There is some clay mineralogical similarity between the clay-sized fraction in the purple rocks and the clay-sized fraction in the overlying purple soils (Fig. 5). The clay fractions in the red purple soils developed from the rocks of the Cretaceous Jiaguan Formation(K_2_*j*) and in Chengqiangyan Group (K_1_*c*) are dominated by chlorite (2*θ* at 6.22°, 12.38°, 25.01°), vermiculite (2*θ* at 6.14°, 31.84°) and illite (2*θ* at 8.84°, 17.73°, 26.76°) and by montmorillonite (2*θ* at 5.85°) and illite (2*θ* at 8.84°, 17.73°, 26.76°), respectively (Fig. 5(a,b)). The calcareous purple soils overlying the thick purplish red mudstone and purplish red sandstone of the Jurassic Penglaizhen Formation (J_3_*p*) and Suining Formation (J_3_*s*) are characterized by montmorillonite (2*θ* at 5.70°~5.85°), chlorite (2*θ* at 6.22°, 12.38°, 25.01°) and illite (2*θ* at 8.84°, 17.73°, 26.76°) and by montmorillonite (2*θ* at 5.70°~5.85°) and illite (2*θ* at 8.84°, 17.73°, 26.76°), respectively (Fig. 5c,d)). Similar results are observed in the XRD diagrams of the neutral purple soils associated with the Jurassic Ziliujing Formation (J_1-2_*z*) and Shaximiao Formation (J_2_*s*) (Fig. 5(e)-(f)). These results support the idea that the clay minerals in the purple soils were dominantly formed by geological processes related to the purple rocks and only slightly influenced by soil-forming processes.

The colloid quality is dependent on the high silicon content in clay minerals (Table 6). The SiO_2_/Al_2_O_3_ ratio in soil colloids varies from 3.67 to 4.63 and is higher in the brown purple soils and reddish brown purple soils and lower in the dark purple soils. The SiO_2_/Fe_2_O_3_ and SiO_2_/R_2_O_3_ ratios in soil clay minerals range from 12.55 to 17.23 and from 2.84 to 3.65, respectively, and are highly associated with the CEC in the purple soils. The CEC values in the purple soils vary from 15.37 cmol_c_ kg^−1^ to 22.24 cmol_c_ kg^−1^, and the exchangeable cations in the purple soils provide a direct source of nutrients for crop growth.

**Table 6:** SiO_2_/Al_2_O_3_, SiO_2_/Fe_2_O_3_ and SiO_2_/R_2_O_3_ ratios in the clay fractions of the purple soils.

Rock	Soil	*n*	SiO_2_ (%)	Al_2_O_3_ (%)	Fe_2_O_3_ (%)	SiO_2_/Al_2_O_3_	SiO_2_/Fe_2_O_3_	SiO_2_/R_2_O_3_	CEC (cmol_c_ kg^−1^)
K_2_*j*	Red purple soil	32	48.25 ± 3.15	22.36 ± 1.12	10.25 ± 0.75	3.67 ± 0.62	12.55 ± 2.03	2.84 ± 0.57	15.37 ± 1.87
K_1_*c*	Yellowish red purple soil	27	50.77 ± 3.04	20.38 ± 1.85	8.89 ± 0.80	4.23 ± 0.45	15.23 ± 2.26	3.31 ± 0.41	17.26 ± 3.28
J_3_*p*	Brown purple soil	43	57.70 ± 3.84	21.20 ± 1.96	8.93 ± 0.83	4.63 ± 0.79	17.23 ± 2.67	3.65 ± 0.63	22.24 ± 4.91
J_3_*s*	Reddish brown purple soil	70	51.98 ± 3.36	20.15 ± 1.46	8.72 ± 0.78	4.39 ± 0.56	15.90 ± 1.91	3.44 ± 0.46	19.32 ± 4.37
J_2_*s*	Grayish brown purple soil	35	52.86 ± 2.71	21.08 ± 1.37	10.40 ± 0.64	4.26 ± 0.48	13.55 ± 0.96	3.24 ± 0.36	16.15 ± 2.31
J_1__-__2_*z*	Dark purple soil	20	50.88 ± 3.03	21.98 ± 2.09	8.91 ± 0.81	3.94 ± 0.75	15.23 ± 2.37	3.13 ± 0.52	16.52 ± 2.29

Note: CEC, cation exchangeable capacity.

#### Calcium carbonate content and pH value of the soils

The calcium carbonate in the purple soils in the Sichuan Basin mainly originates from the parent rocks, and the calcium carbonate contents vary greatly because of differences in the lithologies of the purple rocks and hydrologic and thermal conditions (Table 7). The carbonate contents of the brown purple soil and reddish brown purple soil are relatively high, with values of 6.10% and 6.36%, respectively, and the pH values are approximately 8.0. The carbonate content of yellowish red purple soil is slightly lower than that of the above two purple soils, with a values of 5.51%, and the pH value is approximately 7.8. The calcium carbonate contents of grayish brown purple soil and dark purple soil are 1.12% and 0.84% respectively, and the pH values are approximately 7.0. Calcium carbonate is not present in red purple soil, which has a pH value of approximately 5.7. The large differences in the calcium carbonate contents and pH values of the purple soils result from the parent material and diagenetic environment of the purple rocks and the hydrologic and water-thermal conditions in which the soil formed.

**Table 7:** Calcium carbonate contents and pH values of the purple soils.

Rock	Soil	*n*	Calcium carbonate content (%)	pH
K_2_*j*	Red purple soil	32	—	5.7 ± 0.3
K_1_*c*	Yellowish red purple soil	27	5.51 ± 1.53	7.8 ± 0.1
J_3_*p*	Brown purple soil	43	6.10 ± 1.78	8.1 ± 0.3
J_3_*s*	Reddish brown purple soil	70	6.36 ± 2.15	8.1 ± 0.2
J_2_*s*	Grayish brown purple soil	35	1.12 ± 0.42	7.0 ± 0.3
J_1__-__2_*z*	Dark purple soil	20	0.84 ± 0.31	7.1 ± 0.4

#### Organic matter and nutrient contents of the soils

The OM contents of the purple soils vary from 10.5 g kg^−1^ to 12.7 g kg^−1^ based on difference in topography, vegetation cover, land use patterns, and soil patterns (Table 8). The OM contents of yellowish red purple soil, grayish brown purple soil and dark purple soil are greater than 12.0 g kg^−1^. The OM contents of the red purple soil and brown purple soil are lower than those of the above three purple soils, with values of approximately 11.0 g kg^−1^ and 11.3 g kg^−1^, respectively. The OM content of reddish brown purple soil is the lowest, with the value of approximately 10.5 g kg^−1^. However, after long-term cultivation and crop plantation, the OM can reach values higher than 15.0 g kg^−1^ ^17^. The OM of the purple soils is easily mineralized and difficult to accumulate under the actively aerobic soil conditions as a result of air-filled pores and fragmented characteristics.

**Table 8:** Organic matter and nutrient contents of the purple soils.

Rock	Soil	*n*	OM	TN	AN	TP	AP	TK	AK
			(g kg^−1^)	(g kg^−1^)	(mg kg^−1^)	(g kg^−1^)	(mg kg^−1^)	(g kg^−1^)	(mg kg^−1^)
K_2_*j*	Red purple soil	32	11.3 ± 3.3	0.65 ± 0.21	56 ± 25	0.30 ± 0.15	4.9 ± 1.6	16.9 ± 4.1	53 ± 19
K_1_*c*	Yellowish red purple soil	27	12.7 ± 4.2	0.82 ± 0.32	74 ± 32	0.28 ± 0.18	4.7 ± 1.3	15.7 ± 3.5	63 ± 22
J_3_*p*	Brown purple soil	43	11.0 ± 2.6	0.76 ± 0.27	63 ± 29	0.94 ± 0.42	5.6 ± 1.9	24.9 ± 5.2	91 ± 33
J_3_*s*	Reddish brown purple soil	70	10.5 ± 2.8	0.76 ± 0.24	55 ± 23	0.52 ± 0.34	3.8 ± 1.1	20.8 ± 4.7	87 ± 30
J_2_*s*	Grayish brown purple soil	35	12.4 ± 3.7	0.76 ± 0.26	56 ± 26	0.51 ± 0.23	6.1 ± 2.2	17.1 ± 4.3	82 ± 27
J_1__-__2_*z*	Dark purple soil	20	12.3 ± 3.5	0.83 ± 0.29	86 ± 35	0.44 ± 0.21	5.1 ± 1.7	15.9 ± 3.8	79 ± 25

Note: OM, organic matter; TN, total nitrogen; AN, available nitrogen; TP, total phosphorus; AP, available phosphorus; TK, total potassium; AK, available potassium.

The purple soil nutrient status is characterized by insufficient nitrogen and abundant phosphorus and potassium (Table 8). The mineral nutrient contents in the purple soils are mainly dependent on the original contents in the parent rocks due to rapid weathering of the purple rocks and accelerating anthropogenic processes. The nitrogen content is generally low in the purple soil, with the TN ranging from 0.65 to 0.83 g kg^−1^ and the AN ranging from 55 mg kg^−1^ to 86 mg kg^−1^. Insufficient nitrogen is a key limiting factor in agricultural production in the Sichuan Basin.

The TP content in the purple soils varies from 0.28 mg kg^−1^ to 0.94 g kg^−1^. The high phosphorus content in the purple soils is due to limited losses during soil formation due to weak pedogenesis. Additionally, calcareous phosphorite, such as apatite, in the purple soils can release phosphorus for plant growth during soil development and anthropogenic maturation. However, the phosphorus in phosphorite in neutral and weak alkali purple soils saturated with calcium carbonate is released very slowly to the soil; hence, the AP in the purple soils is not very high, ranging from 3.8 mg kg^−1^ to 6.1 mg kg^−1^. Although phosphorus is generally considered to be relatively immobile in soils, significant redistribution occurs during pedogenesis^34^. The phosphorus in the acidic purple soils, which have experienced intense eluviation during diagenesis or pedogenesis, has been heavily lost, and the total and available amounts are very low, which has a significant effect on phosphorus fertilization.

Potassium is abundant in the purple soils, especially in the calcareous purple soils. The TK and AK in the purple soils vary from 15.7 to 24.9 g kg^−1^, and from 53 to 91 mg kg^−1^, respectively, and therefore can meet the needs of crop growth. In the purple soils, potassium exists in primary minerals, such as feldspar and mica, and secondary clay minerals, such as illite, and can be released during physical or biochemical weathering processes. Hence, TK is slowly converted to AK for plant growth.

## Discussion

### Obvious inheritance from parent rocks

The purple soils, whether natural or agriculturally modified in hilly or mountainous areas, developed from the purple sedimentary rocks that were deposited and diagenetically altered in the Sichuan Basin in the Jurassic and Cretaceous periods and are characterized by obvious characteristics, such as their inherited color, particle size distribution, mineralogy, chemical composition, pH value, OM content and nutrient content.

First, as evidenced in the soil profile (Fig. 4), the colors of the purple soils are highly consistent with those of the parent rocks. In other words, there is little or no difference between the color of the soil and that of the parent rocks. This pedogenetic phenomenon is different from that associated with the red soil and yellow soil influenced by a strong material cycle and chemical weathering in the subtropical zone of China. The purple soils’ occurrence and color originate from the parent rocks, which formed in various paleoenvironments, instead of originating as products of recent soil-forming processes^17^. The diagenesis occurring in the Jurassic and the Cretaceous periods in the Sichuan Basin was driven by alternating arid and humid environments. The color of the soil developed from the purple rocks in Sichuan Basin is largely related to the presence of iron and manganese oxides^35^. The purple tone varies from yellow to red or purple with increasing crystalline iron oxides (hematite) in the rocks, and a higher content of manganese oxides in the rocks produces a darker soil color. In the Sichuan Basin, the term “purple soil” refers not only to the soil color but also to the mean properties and nature of the soils, such as a high degree of physical weathering and pedogenetic processes, a strong resistance to chemical weathering, and a high fertility. Hence, the purple color is one of the most important indexes for soil classification.

Additionally, the grain size compositions of the purple rocks and purple soils (Tables 1 and 5) indicates that the grain size compositions of the soils developed on different purple rocks are consistent with those of their parent rocks. On the one hand, in the purple rocks, the sand contents range between 35.83% and 58.69% and tend to be higher in the red purple rocks (58.69%), grayish brown purple rocks (48.11%) and dark purple rocks (47.54%) and lower in the other purple rocks (35.83–39.38%). Additionally, the clay contents of the purple rocks follow the opposite pattern: the red purple rocks (14.56%) and grayish brown purple rocks (15.33%) have much lower clay contents than the other rocks (17.38–18.79%). On the other hand, the sand contents of the purple soils derived from rocks deposited in different geological periods range between 36.66% and 61.28% and exhibit the following order: red purple soils (61.28%) > grayish brown purple soils (51.26%) > dark purple soils (47.60%) > other purple soils (36.66–38.97%). Similarly, the clay contents of the red purple soils (16.38%) and grayish brown purple soils (18.36%) are much lower than those of the other soils (21.81–23.32%). The above results are consistent with previous research results^23^. Therefore, the grain size characteristics of purple soils, unlike other soils such as red soils^31^, are dependent on the lithofacies of the parent rocks.

Millot (1970) distinguished three principal processes to account for the genesis of clay minerals: (1) inheritance from parent materials, (2) transformation of other clay minerals, and (3) precipitation from the soil solution^36^. The clay-sized fraction in the purple soils is generally dominated by illite, vermiculite, chlorite, and montmorillonite, with little quartz and with or without kaolinite because of the low degree of weathering. There is obvious clay mineralogical similarity between the clay-sized fractions in the purple rocks and the clay-sized fractions in the overlying purple soils (Fig. 5). In addition, the chemical compositions of the clay fractions in the soils are consistent with those in their parent rocks (Tables 2 and 6). These results prove that the mineralogical characteristics and chemical compositions of the purple soils mainly result from inheritance from the parent materials.

In addition, the pH values, OM contents and nutrient contents of the purple soils are closely related to those of the parent rocks. Acidic purple rocks develop acidic soils, and calcareous purple rocks develop calcareous purple soils. However, the pH values of the purple rocks are higher than those of the purple soils. In the early Jurassic period, with a humid climate and dense thick vegetation, dark lacustrine sedimentary rocks were mainly deposited and diagenetically altered and were rich in OM and heavily decomposed substances. These rocks are purple, dark purple, and grayish purple in color and correspond to the Jurassic Shaximiao Formation (J_2_*s*) and Ziliujing Formation (J_1-2_*z*). In the middle-late Jurassic and the Cretaceous periods, with arid and hot environments and few plants, less OM was deposited, thereby impacting the diagenesis of red sedimentary rocks. These rocks are reddish brown, purplish red, and dark purplish in color and correspond to the Jurassic Suining Formation (J_3_*s*). The purple rocks and purple soils are characterized by insufficient nitrogen and abundant phosphorus and potassium. The purple sedimentary rocks are compositionally complex and variable and are generally regarded as nutrient reservoirs because of their high nutrient contents, especially phosphorus and potassium^17^. The mineral nutrient contents in the purple soils are mainly dependent on the nutrient contents in their parent rocks due to rapid weathering of purple rocks and accelerated anthropogenic processes.

### Rapid physical weathering and pedogenetic processes

Under natural conditions, it takes a long time for soils to develop on sandy rocks, limestone, and granite. For instance, on limestone, the development of a 1 cm soil layer is expected to take approximately 2.5–8.5 kyr^37^. Without erosion, granitic bedrock may need more than 230 kyr or more than 1340 kyr to form 7 m of weathered regolith, and the rate depends on the relative contribution of chemical and physical weathering to the density decrease^38^. The pedogenesis of purple soils is characterized by rapid physical pedogenetic processes associated with the rocks, parent material, and soil^3,39^. The pedogenetic capacities of the purple rocks under natural conditions, i.e., 24.84%, are higher than those of other types of sedimentary rocks^3^. That is, it takes only approximately 5 years to transform the purple rocks into soil particles. The characteristics of the purple soils are highly dependent on their parent rocks. Most purple rocks in the Sichuan Basin are lacustrine deposits, and their material sources are mainly colloids or fine-grained material associated with fossil weathering crust or paleosols.

These terrigenous deposits were transformed into sedimentary rocks under high pressure and were cemented by clayey, siliceous, ferruginous, and calcareous cement. When the sedimentary rocks are exposed at the earth’s surface, the cement is easily dissolved or corroded through the influence of air (oxygen) and acid or alkaline solutions resulting in the collapse or fragmentation of the sedimentary rocks. The purple rocks are composed of interbedded sandstones and mudstones, and the mudstones are easily weathered, thereby accelerating the weathering processes in the purple rocks. The purple rocks are more complex in terms of mineral composition than other types of rocks. The mineral particles larger than 0.02 mm mainly consist of primary minerals, such as quartz, feldspar, and mica, and those less than 0.0001 mm mainly consist of secondary minerals, such as illite, montmorillonite, vermiculite, and chlorite (Fig. 5). In response to changes in temperature and shifts between dry and wet conditions, the rocks experienced heterogeneous swelling and shrinkage and are heavily cracked, and resulting in rock fragments less than 2 mm in diameter, which are referred to as soil particles.

Disturbances, either natural or artificial, are common and helpful in determing the genesis and distribution of soils^40^. The purple rocks have been directly broken up by dynamite or manually with hammers, shovels, and hoes into rock fragments or gravel, in which crops can be planted immediately. It takes more than 10 years or purple rock saprolite transform into the fertile agricultural soils by anthropogenic activity, such as tillage, crop planting, straw application, and land reclamation^3,17^. Soil desertification in the Sichuan Basin has not taken place as a result of rapid physical weathering of purple rocks and associated pedogenesis, which have balanced erosion-related soil and grus losses^17,18,39^. Accordingly, agricultural production in Sichuan Basin has been maintained or has developed further.

### Slow chemical pedogenetic process

The purple soil formation is characterized by the simultaneous eluviation of base exchangeable ions in soils and their compensation via the weathering of purple rocks. The physical and chemical weathering processes occur simultaneously in purple soils^16,39^. Under humid subtropical conditions, the base exchangeable ions, such as Ca^2+^, Mg^2+^, K^+^, and Na^+^, are heavily eluviated in the purple soils. However, the weathering of the parent rocks can sufficiently supply the soils with base exchangeable ions so that the concentration of base exchangeable ions in the soils remains high.

Decalcification occurs in the pedogenetic process of purple soils, and the mineral and chemical compositions of the rocks and their developed soils are similar^39^. Therefore, the weathering intensity in the purple soils is weak, and the soil-forming chemical process is slow. Most purple rocks are terrestrial deposits with chemical or physiochemical characteristics that formed under arid and humid climatic conditions^17^. Over geologic time (millions of years to more than 100 million years), the amorphous iron oxides associated with the mineral particles gradually crystallized, making the sediments resistant to chemical weathering. The active surfaces of the mineral particles in the purple rocks have been aged. The calcium carbonates present in the purple rocks has greatly decelerated the decalcification and desilication of the soils because of the weakly alkaline hydrologic environment. The intense erosion of the surface soils every year is balanced by rapid physical weathering and pedogenetic processes. Therefore, chemical weathering is limited during the early stages of soil formation.

Under undisturbed conditions, soil is a continuum formed by the interaction of parent material, vegetation and time^40^. However, this continuity may be suddenly interrupted by natural or artificial high-intensity disturbances, which can trigger vegetation changes and disrupt soil evolution, or by low intensity perturbations affecting the dynamics of OM^40,41^. In fact, the long-term effects of the evolution of a large quantity of OM on soil aggregation dynamics and structural characteristics are not well known. Grosbellet *et al*. (2011) found that the soil structure can be improved by the degradation of coarse OM^42^. For calcareous purple soils or parent materials developing in a relatively flat position under calm conditions over a long period of time, their calcium carbonate contents could be strongly eluviated under a subtropic climate with abundant precipitation. Furthermore, the eluviation of calcium carbonate is accelerated by some types of microtopography (potentially resulting in pooling because of a poor irrigation strategy), agricultural practices (acidic fertilizer), and environmental deterioration (acidic rain).

## Conclusion

This study was conducted in the Sichuan Basin, which is mainly covered by Triassic to Cretaceous red and purple rock series. The results from this study indicate that the soil profiles contain no clear stratification but do contain rock fragments. The particle size contents of the soils are consistent with those of their parent purple rocks. The clay-sized fractions in the purple rocks and purple soils are generally dominated by illite, vermiculite, chlorite, and montmorillonite with little quartz and with or without kaolinite. In addition, the chemical compositions, pH values, OM contents and nutrient contents of the purple soils are closely related to those of the parent rocks. The diagenetic environment determined the lithology of the purple rocks and the lithology of the purple rocks determined the pedogenic characteristics of purple soil to some extent. The purple soils are characterized by rapid physical weathering and pedogenetic processes, and slow chemical pedogenetic processes. The rapid physical weathering produces a high rate of purple soil formation but the slow chemical weathering places the purple soil in the early stage of soil formation. This study can help with understanding the lithological features of the rocks and their effects on the pedogenesis of soils, and the results will promote the sustainable agricultural development in the Sichuan Basin in southwestern China.

## Supplementary information

Dataset 1

## Data Availability

The original data can be obtained from the authors upon reasonable request.

## References

[1] Repe, B., Simončič, P. & Vrščaj, B. Factors of Soil Formation in *The Soils of Slovenia* (eds Vrščaj, B., Repe, B. & Simončič, P.) 19–60 (Springer Netherlands, 2017).

[2] Jenny, H. *Factors of Soil Formation: A System of Quantitative* Pedology. (Dover Publications, 1994).

[3] Wei C, Ni J, Gao M, Xie D, Hasegawa S (2006). Anthropic pedogenesis of purple rock fragments in Sichuan Basin, China. Catena.

[4] Amundson R, Jenny H (1997). Thinking of biology: On a state factor model of ecosystems. Bioscience.

[5] Yu F, Hunt AG (2018). Predicting soil formation on the basis of transport-limited chemical weathering. Geomorphology.

[6] Sumner, M. E. *Handbook of Soil Science*. (CRC Press, 2000).

[7] Bardgett, R. D. *The biology of soil: a community and ecosystem approach*. (Oxford University Press, 2005).

[8] Daher M, Schaefer CEGR, Fernandes Filho EI, Francelino MR, Senra EO (2019). Semi-arid soils from a topolithosequence at James Ross Island, Weddell Sea region, Antarctica: Chemistry, mineralogy, genesis and classification. Geomorphology.

[9] Targulian VO, Krasilnikov PV (2007). Soil system and pedogenic processes: Self-organization, time scales, and environmental significance. Catena.

[10] Lin H (2010). Linking principles of soil formation and flow regimes. J. Hydrol..

[11] Almond, P. C. Soils and geomorphology of a lowland rimu forest managed for sustainable timber production. PhD thesis, *Lincoln University* (1997).

[12] Almond PC, Tonkin PJ (1999). Pedogenesis by upbuilding in an extreme leaching and weathering environment, and slow loess accretion, south Westland, New Zealand. Geoderma.

[13] Phillips JD (2004). Geogenesis, pedogenesis, and multiple causality in the formation of texture-contrast soils. Catena.

[14] Mirabella A, Vecchio G, Risi B (1996). Caratterizzazione mineralogica dei suoli su granito e micascisto in Sila Grande. Calabria Verde.

[15] Gracheva R (2011). Formation of soil diversity in the mountainous tropics and subtropics: Rocks, time, and erosion. Geomorphology.

[16] Wei, C., Xie, D. & Che, F. On particle size distribution of purple soil and its chemical composition in southern Sichuan basin. *Journal of Southwest Agricultural University*, 425–428 (1989).

[17] He, Y. *Purple Soils in China (2)*. (Chinese Science Press, 2003).

[18] Zhu B, Wang T, You X, Gao MR (2008). Nutrient release from weathering of purplish rocks in the Sichuan Basin, China. Pedosphere.

[19] Wei C (2008). Soil Aggregation and Its Relationship with Organic Carbon of Purple Soils in the Sichuan Basin, China. Agricultural Sciences in China.

[20] Zhong S, Zhong M, Wei C, Zhang W, Hu F (2016). Shear strength features of soils developed from purple clay rock and containing less than two-millimeter rock fragments. Journal of Mountain Science.

[21] Zhang X (2006). A preliminary assessment of the potential for using Pb-210(ex) measurement to estimate soil redistribution rates on cultivated slopes in the Sichuan Hilly Basin of China. Catena.

[22] Zhang XB, Zhang YY, Wen AB, Feng MY (2003). Assessment of soil losses on cultivated land by using the Cs-137 technique in the Upper Yangtze River Basin of China. Soil & Tillage Research.

[23] Zhong S, Bo L, Wei C, Hu F (2015). The rock fragments (<2 mm) and their action mechanism on the shear strength of purple mudstone-developed soils. Scientia Agricultura Sinica.

[24] Zheng, C. The runoff and its adjustment on slope land of Sichuan Basin. Master thesis, *Southwest University* (2008).

[25] Sichuan Bureau of Geology and Mineral Resources. *Geology of Sichuan province*. (Geological Publishing House, 1991).

[26] National Energy Administration of China. *Analysis method for particle size of clastic rocks*. (Petroleum Industry Press, 2009).

[27] Agrochemistry Committee of Soil Science Society of China. *Conventional Methods of Soil and Plant Analysis*. (Science Press, 1983).

[28] Page, A. L. *Methods of Soil Analysis: Part 2. Chemical and microbiological properties*. (Am. Soc. of Agron., Inc., 1982).

[29] Brindley, G. W. & Brown, G. *Crystal Structures of Clay Minerals and Their XRD Identification*. (Mineralogical Society, 1980).

[30] Sichuan Province Agriculture Bureau. *Soils in Sichuan*. (Sichuan Science and Technology Press, 1995).

[31] Wilson, M. J., He, Z. & Yang, X. *The red soils of China: their nature, management, and utilization*. (Kluwer Academic Publishers, 2004).

[32] Dane, J. H. & Topp, G. C. *Methods of Soil Analysis: Part 4. Physical Methods*. (Soil Science Society of America, 2002).

[33] Wilson MJ (1999). The origin and formation of clay minerals in soils: past, present and future perspectives. Clay Min..

[34] Letkeman LP, Tiessen H, Campbell CA (1996). Phosphorus transformations and redistribution during pedogenesis of western Canadian soils. Geoderma.

[35] He, Y., Sato, Y. & Hotan, S. Study on soil color of purple soils. *Chinese Journal of Soil Science*, 247–250 (1990).

[36] Millot, G. *Geology of Clays: weathering*, sedimentology, geochemistry. (Masson et Cie, 1970).

[37] Wang SJ (1999). Preliminary study on weathering and pedogenesis of carbonate rock. Sci. China Ser. D-Earth Sci..

[38] Dethier DP, Lazarus ED (2006). Geomorphic inferences from regolith thickness, chemical denudation and CRN erosion rates near the glacial limit, Boulder Creek catchment and vicinity, Colorado. Geomorphology.

[39] Du J, Luo Y, Zhang W, Xu C, Wei C (2013). Major element geochemistry of purple soils/rocks in the red Sichuan Basin, China: implications of their diagenesis and pedogenesis. Environmental Earth Sciences.

[40] Scalenghe R, Bonifacio E, Celi L, Ugolini FC, Zanini E (2002). Pedogenesis in disturbed alpine soils (NW Italy). Geoderma.

[41] Richter DD, King KS, Witter JA (1989). Moisture and nutrient status of extremely acid Umbrepts in the Black Mountains of North Carolina. Soil Sci. Soc. Am. J..

[42] Grosbellet C, Vidal-Beaudet L, Caubel V, Charpentier S (2011). Improvement of soil structure formation by degradation of coarse organic matter. Geoderma.

